# BK Polyomavirus MicroRNA Levels in Exosomes Are Modulated by Non-Coding Control Region Activity and Down-Regulate Viral Replication When Delivered to Non-Infected Cells Prior to Infection

**DOI:** 10.3390/v10090466

**Published:** 2018-08-30

**Authors:** Francesco Martelli, Zongsong Wu, Serena Delbue, Fabian H. Weissbach, Maria Chiara Giulioli, Pasquale Ferrante, Hans H. Hirsch, Simone Giannecchini

**Affiliations:** 1 Department of Experimental and Clinical Medicine, University of Florence, I-50134 Florence, Italy; martelli.francesco85@gmail.com (F.M.); maria.chiara2591@gmail.com (M.C.G.); 2 Transplantation & Clinical Virology, Department Biomedicine (Haus Petersplatz), University of Basel, CH-4003 Basel, Switzerland; zongsong.wu@unibas.ch (Z.W.); fabian.weissbach@unibas.ch (F.H.W.); 3 Department of Biomedical, Surgical and Dental Sciences, University of Milan, I-20100 Milano, Italy; serena.delbue@unimi.it (S.D.); pasquale.ferrante@unimi.it (P.F.); 4 Infectious Diseases & Hospital Epidemiology, University Hospital Basel, CH-4003 Basel, Switzerland

**Keywords:** *polyomavirus*, *non-coding control region*, microRNA, exosomes, persistence, immunosuppression, *BK virus*

## Abstract

In immunosuppressed patients, *BKPyV*-variants emerge carrying rearranged non-coding control-regions (*rr-NCCRs*) that increase early viral gene region (EVGR) expression and replication capacity. *BKPyV* also encodes microRNAs, which have been reported to downregulate EVGR-encoded large T-antigen transcripts, to decrease viral replication in infected cells and to be secreted in exosomes. To investigate the interplay of *NCCR* and microRNAs, we compared archetype- and *rr-NCCR-BKPyV* infection in cell culture. We found that laboratory and clinical *rr-NCCR-BKPyV*-strains show higher replication rates but significantly lower microRNA levels than archetype virus intracellularly and in exosomes. To investigate whether *rr-NCCR* or increased EVGR activity modulated microRNA levels, we examined the (*sp1-4*)*NCCR-BKPyV*, which has an archetype *NCCR*-architecture but shows increased EVGR expression due to point mutations inactivating one Sp1 binding site. We found that microRNA levels following (*sp1-4*)*NCCR-BKPyV* infection were as low as in *rr-NCCR*-variants. Thus, *NCCR* rearrangements are not required for lower miRNA levels. Accordingly, Sp1 siRNA knock-down decreased microRNA levels in archetype *BKPyV* infection but had no effect on (*sp1-4*)- or *rr-NCCR-BKPyV*. However, *rr-NCCR-BKPyV* replication was downregulated by exosome preparations carrying *BKPyV*-microRNA prior to infection. To explore the potential relevance in humans, urine samples from 12 natalizumab-treated multiple sclerosis patients were analysed. In 7 patients, *rr-NCCR-BKPyV* were detected showing high urine *BKPyV* loads but low microRNAs levels, whereas the opposite was seen in 5 patients with archetype *BKPyV*. We discuss the results in a dynamic model of *BKPyV* replication according to *NCCR* activity and exosome regulation, which integrates immune selection pressure, spread to new host cells and *rr-NCCR* emergence.

## 1. Introduction

*BK polyomavirus (BKPyV)* is one of more than 10 human *polyomaviruses* (*HPyV)* and it infects >90% of the general human population without ill effects [1,2,3]. *BKPyV* causes nephropathy or haemorrhagic cystitis in immunocompromised patients, particularly after kidney or allogeneic hematopoietic cell transplantation [4,5]. Seroprevalence data indicate that *BKPyV* transmission occurs in early childhood [6] via the oral or respiratory route [7]. Subsequently, *BKPyV* reaches the renourinary tract, presumably by primary viremia [7], where the virus establishes a latent infection [8]. Asymptomatic low-level *BKPyV* shedding has been demonstrated in 10% of *BKPyV* IgG-seropositive healthy blood donors [9] providing evidence of immune escape in adult immunocompetent hosts. Antibody levels decline during adult life [9,10] unless significant re-exposure occurs which includes immunocompromised patients [11,12,13,14]. In immunocompromised individuals, however, urinary *BKPyV* shedding is more frequent, often with urine viral loads of exceeding 7 log10 copies/mL that become apparent as “decoy cell” shedding [2,15,16,17,18]. In kidney transplant or allogeneic hematopoietic cell transplant patients, high-level *BKPyV* replication precedes nephropathy or haemorrhagic cystitis, respectively [4,5]. Increased *BKPyV* reactivation rates have also been observed in other solid organ transplant recipients and in *HIV*/AIDS patients [19,20,21]. However, the molecular details of regulating *BKPyV* replication in immunocompetent individuals and the relevance for disease progression are not well understood.

*BKPyV* has a double-stranded DNA genome of approximately 5 kb, which can be divided into: (i) the early viral gene region (*EVGR*) encoding the small and the large T-antigen; (ii) the late viral gene region (*LVGR*) encoding the capsid proteins Vp1, Vp2, Vp3, agnoprotein and microRNAs; and (iii) the *non-coding control region* (*NCCR*) [2,22]. The *NCCR* harbours the origin of genome replication *ori* and promoter/enhancer with DNA-binding sites for transcription factors mediating the secondary host cell specificity [23], as well as the timing and course of *EVGR*-expression, viral DNA-replication and *LVGR*-expression [2].

*BKPyV* sequences commonly found in the urine of healthy persons have an archetype *NCCR* architecture of sequence blocks arbitrarily denoted O-P-Q-R-S [2,9]. In immunocompromised patients with *BKPyV*-disease, viral variants with rearranged *NCCR (rr-NCCR*) architecture have been shown to emerge as majority species and are associated with disease severity [24]. In these patients, archetype *NCCR-BKPyV* is still found in urine but molecular cloning has demonstrated the presence of a viral *quasi*-species with co-existing *rr-NCCR* minority species [25], which may be an indicator of imminent pathology [26]. In vitro studies and in vivo observations support the view that *rr-NCCRs* confer a higher replicative activity in vitro but which depends on the lack of cellular immune functions in vivo [24]. A similar link between *rr-NCCR* and disease was also observed for *JCPyV* in *HIV*/AIDS patients with progressive multifocal leukoencephalopathy [27,28], or for *HPyV-7* and *HPyV-9* [23]. Together, the data suggest that *HPyV-NCCR* rearrangements arising in immunocompromised patients are not only a surrogate marker of long-standing immunologically uncontrolled replication but also represent a virulence determinant of activated *EVGR* expression and increased replication capacity causing disease.

Given the complex diversity of *NCCR* rearrangements that affect various transcription factor binding sites as well as the overall architecture, a systematic study of inactivating specific transcription factor binding sites by point mutation was conducted, which maintained the linear archetype architecture of *BKPyV-NCCR* [29]. Of note, mutations inactivating the *Sp1* site located proximal to the *LVGR* promoter termed *SP1-4* resulted in a phenotype functionally equivalent to *NCCR* rearrangements (group 1) and which had been identified in patients with *BKPyV* disease [29]. Intriguingly, a similar, albeit low-affinity Sp1 binding site *SP1-2* has been located upstream of the EVGR promoter, the inactivation of which (e.g., *sp1-2*) decreased EVGR expression and replication (group 3) [29]. Further mutational dissection of the *BKPyV-NCCR* as well as electrophoretic mobility shift assays and chromatin immunoprecipitation analysis revealed that EVGR-expression involves a classic inducible TATA-box promoter. Upstream, the *EVGR* promoter partially overlaps with a constitutive housekeeping gene-type *LVGR* promoter using a TATA-like box in the opposite orientation, in which a high-affinity *SP1-4* sites acts as a central switch of bidirectional gene expression [30]. *sp1-4* point mutations inactivating Sp1 binding cause constitutive activation of *EVGR* expression and increased viral replication without NCCR rearrangements [30].

Besides the *NCCR*, another layer of regulating EVGR expression has been described at the posttranscriptional level mediated by microRNAs (miRNAs) miRNA-B1-3p and miRNA-B1-5p encoded in the distal *LVGR* [22,31,32,33]. Similar to other *PyVs* [31,33,34,35], *BKPyV* miRNAs are short noncoding RNAs, which target large T-antigen transcripts and thereby down-regulate viral replication [32,36,37]. This posttranscriptional safeguard has been implicated in the escape from large T-antigen-specific cytotoxic T-cells [34], which have recently been linked to the effective curtailing of *BKPyV* replication in kidney transplant patients [38,39]. The regulation of miRNAs expression is subject of ongoing studies, which may involve sequences close to the miRNA gene as well as the *NCCR* [22,40,41]. Moreover, miRNAs of *BKPyV* and the closely related *JCPyV* miRNAs have been detected in blood, urine and cerebrospinal fluid samples, often together with the corresponding viral loads with few cases reporting the nature of the *NCCR* structures [42,43]. It has been suggested that urinary exosomes associated *BKPyV*-miRNA may be a surrogate marker for *BKPyV* pathology [44]. Thus, the association between miRNAs and exosomes has also raised questions about their regulatory potential in non-infected neighbouring cells [43,45,46].

Since the *NCCR* and the miRNA represent two different, formally independent modalities of regulating *BKPyV* replication at the transcriptional and post-transcriptional level, respectively, we examined the interplay of *NCCR* architecture and miRNAs expression following *BKPyV* infection. Imperiale and colleagues reported that *BKPyV* miRNA levels were low following infection with laboratory strains carrying *rr-NCCR*. In a first step, we therefore compared miRNA levels in archetype and different laboratory- and patient-derived *rr-NCCR-BKPyV* variants in cell culture. We also examined the miRNA levels seen with the (*sp1-4*)*NCCR-BKPyV* and (*sp1-2*)*NCCR-BKPyV*, which, as outlined above, have an archetype architecture but show increased and decreased EVGR expression and viral replication, respectively. The role of Sp1 was further evaluated by siRNA-*SP1* known-down. To investigate whether or not *rr-NCCR-BKPyV* replication could still be down-regulated by mircoRNA, we transferred exosomes carrying *BKPyV*-microRNA cargo onto uninfected cells prior to infection. Finally, we explore the potential relationship of *BKPyV* load, *NCCR* architecture and miRNA levels in vivo in urines from multiple sclerosis patients treated with natalizumab. We then attempt to integrate the results in a dynamic model of transcriptional and posttranscriptional regulation and discuss the potential implications in immunocompetent and immunosuppressed hosts, favouring archetype and rearranged *NCCR-BKPyV*, respectively.

## 2. Materials and Methods

### 2.1. Urine Sampling

The urine samples used in this study were obtained from 12 relapsing-remitting multiple sclerosis patients undergoing intravenous natalizumab treatment at the Multiple Sclerosis Center, Neurological Institute Mondino, Pavia, and had enrolled for a previous study [47]. None of them developed symptoms or signs of polyomavirus-associated diseases at presentation or during the follow-up. The study was approved by the local institutional review board, “Fondazione Istituto Neurologico Mondino”, Pavia, Italy (n. 101MS326). Human samples were taken after obtaining the informed consent from the patients or control subjects in accordance with the tenets of the Declaration of Helsinki.

### 2.2. Cell Cultures and BkPyV Molecular Clones

African green monkey kidney, SV40-transformed Cos-7 cells line (CRL1651, ATCC, Manassas, VA, USA [48]) were grown in Dulbecco modified Eagle’s medium (DMEM) supplemented with 10% foetal bovine serum (FBS, SIGMA, Milan, Italy). Primary human renal proximal tubule epithelial cells (RPTECs, PCS-400-010, ATCC, Manassas, VA, USA) were grown in an epithelial cell medium supplemented with epithelial cell growth supplement and 2% FBS (ScienceCell Research Laboratory, Carlsbad, CA, USA). *BKPyV* strains were derived from molecular clones carrying archetype *NCCR* (clone *WW1.4*) [24], a *rr-NCCR* laboratory strain (Dunlop, Akron, OH, USA) [49], clinical rr-NCCR variants having deletions in the Q- and the R-block (clone *del5.3*, *del15.10*) [24,29] and the archetype *NCCR* variants carrying point mutations of Sp1 binding sites in the Q-block (clone *sp1-4*) or in the P-block (clone *sp1-2*) [29,30].

### 2.3. BKPyV Infection of Cos-7 and RPTECs

Transfection of *BKPyV* genomic DNA into Cos-7 cells was performed at 90–95% confluence in 6-well plates using Lipofectamine 2000 (Invitrogen, Carlsbad, CA, USA) according to the manufacturer’s instructions. At 6 h posttransfection, the medium was replaced by DMEM containing 10% FBS. At 14 days posttransfection, Cos-7 cells were harvested by scraping off cells in 1/10 of the cell culture supernatant. Virus was released by 3 cycles of freeze-thawing of the cells and centrifugation at 800 *g* for 5 min. For the infection experiments, Cos-7 and RPTECs were seeded at 7.5 × 10^5^ cells and 3.12 × 10^5^ cells per well in 6-well plate with 2 mL of DMEM (10% FBS) or supplemented EpiCM medium (2% FBS). After 24 h at a confluence of approximately 70%, Cos-7 and RPTECs were exposed to 500 microliters of the corresponding virus preparations (MOI 1) obtained from Cos-7 cells after transfection at 37 °C for 2 h followed by removal and replacement with DMEM (10% FBS) or supplemented EpiCM medium (0.5% FBS). Cells and supernatant were harvested at 12, 24, 48 and 72 h post infection. Titration revealed that approximately 5–10 × 10^6^ genome copies of *BKPyV*-Dunlop genomes on 50,000 RPTECs or Cos-7 cells at infection typically elicits a multiplicity of infection of 1 after 48 h according to fluorescent focus forming units using staining for large T-antigen; H.H. Hirsch and M. Wernli, unpublished results and see References [50,51].

### 2.4. Plasmid Exosomes BKPyV miRNA Expression in Cos-7 Cells

*BKPyV*-miRNAs were inserted into the pcDNA 6.2GW/EmGFP-miR (Invitrogen, Carlsbad, CA, USA) vectors and cloned according to the manufacturer’s instructions. To this end, specific oligonucleotide pairs were designed on the *BKPyV* miRNA mature sequence (5′TGCTGATCTGAGACTTGGGAAGAGCATTTTTGGCCACTGACTGAATGCTCTTCCCATCTCAGAT3′ forward oligonucleotide and 5′CCTGATCTGAGATGGGAAGAGCATTCAGTCAGTGGCCAAAAATGCTCTTCCCAAGTCTCAGATC3′ reverse oligonucleotide; *BKPyV*-miRNA mature sequence is underlined below the sequences shown). Next, the corresponding expression vectors pcDNA6-BK-miRNA (10 µg) were transfected into Cos-7 cells (7.5 × 10^5^ cells per well in 6-well plate) using lipofectamine 2000 (Invitrogen, Carlsbad, CA, USA). *BKPyV*-miRNA expression vectors system (encodes for the green fluorescence protein GFP) efficiency was assessed at 24h after transfection based on the number of GFP-positive cells, as determined by flow cytometry. In subsequently experiments exosomes carrying *BKPyV*-miRNA were purified at 24 h, 48 h and 72 h from transfected Cos-7 cells and were quantified. The sequence of the miRNA inserts was confirmed by sequencing.

### 2.5. Exosome Enriched Vesicles Extraction

The exosome-enriched vesicles were isolated starting from 250 µL of cells supernatant collected by prior centrifugation at 14,000 *g* for 20 min, using the exosome-specific extraction kit (Norgen, Thorold, ON, Canada), following the manufacturer’s protocols. Characterization of the exosome-preparation was done by demonstrating the presence of 112 nm vesicles present at a density of a mean total particles concentration of 10^7^/mL. Further characterization was achieved by western blot, detecting three markers of exosome tetraspanin/CD63, CD81 and Annexin II (Supplement Figure S1). Before analysis, exosome-enriched vesicles were treated with RNase and DNase to remove miRNA and DNA not protected inside the exosomes.

### 2.6. Exosome Addition and Anti BKPyV-miRNA Inhibition in Cos-7 Cells

Exosomes carrying *BKPyV*-miRNA (10^3^ copies) were added to the Cos-7 cells (7.5 × 10^5^ cells per well in 6-well plate) and used to test the effect on subsequent viral infection. Where indicated, the exosomes were added to Cos-7 cells in the absence or presence of inhibitory specific antago-BK-miRNA-5p (Inh-BK-miRNA, a phosphorothioate oligonucleotide with the sequence complementary to the BK-miRNA) synthetic molecule, which had been transfected into Cos-7 cells using a 5 µM final concentration, together with lipofectamine 2000 (Life Technologies, Foster City, CA, USA). Unrelated miRNA (Unrel-miRNA, a phosphorothioate oligonucleotide with an unrelated sequence) synthetic molecule, was used as control.

### 2.7. BKPyV DNA Quantification

Viral DNA was extracted from 0.15 mL of urine, from 2 × 10^4^ cells and cell supernatant of tissue cultures with the High Pure PCR Template Preparation kit (Roche, Basel, Switzerland). DNA extracted was subjected to quantitative real-time PCR (qPCR) assays using primer and probe targeting Vp1 gene (BKVPf forward primer 5′-AGTGGATGGGCAGCCTATGTA-3′, BKVPr reverse primer 5′-TCATATCTGGGTCCCCTGGA-3 and BKVPp TaqMan MGB probe labelled with VIC VIC-5′ AGGTAGAAGAGGTTAGGGTGTTTGATGGCACAG 3′) (Life Technologies, Foster City, CA, USA). Each reaction was carried out with negative controls (no template) and DNA standards (diluted to contain 10^1^–10^6^ copies per millilitre) of a plasmid containing the *BKPyV* molecular clone. The lowest limit of detection of the assay was 10 copies per millilitre of urine and micrograms of total DNA [52].

### 2.8. BKPyV Pre-miRNA and Mature miRNA Quantification

Total RNA was isolated from 2.0 × 10^6^ Cos-7 and RPTECs cells using the mirVana miRNA isolation Kit (Ambion, Foster City, CA, USA), and from exosomes contained in 250 µL of cell-free supernatant that had previously been centrifuged at 14,000 *g* for 20 min using an RNA exosome-specific circulating extraction kit (Norgen). The miRNA expression was measured and quantified with a specific pre-miRNA and mature bk-miRNA-5p quantitative stem-loop RT-PCR MiRNA assay whose primers were designed on the specific region of the *BKPyV* WW clone (Life Technologies, Foster City, CA, USA) according to the manufacturer’s protocol. Each reaction was performed in triplicate using 10 ng of extracted RNA, including negative controls (no template) and synthesized oligonucleotides as standards (diluted to contain 10^1^–10^6^ copies). The lowest detection limit of the assay was 10 copies/ng of RNA. The assay was specific and reproducible, as demonstrated in preliminary experiments using a *BKPyV* oligonucleotide standard (with <0.5 Ct value inter-assay variation) and observing no amplification of unrelated oligonucleotide targets.

### 2.9. siRNA knock-Down of *Sp1* and Immunoblotting

Cos-7 cells were transfected in a 6-well plate using per well 2 µg of siRNA targeting Sp1 (SP1-siRNA, Mission esiRNA SIGMA), or 2 µg of siRNA scrambled form (Scr-siRNA), or mock-treated without siRNA added (mock). At 24 h post-transfection, Cos-7 cells were infected at multiplicity of infection (MOI) of 1, using BKPyV-infectious supernatant obtained after transfection of Cos-7 cells. BK-miRNA-5p expression in cells and presence in exosomes enriched vesicles were measured at 48 h post infection. Immunoblots were used to compare Sp1 expression and cytochrome P450 as control. Briefly, cell extracts were prepared from Cos-7 cells at 48 h after transfection of SP1-siRNA, Scr-siRNA and mock-treatment and 15 µg of extract were analysed by 10% SDS-PAGE, transferred onto nitrocellulose membrane (BIO-RAD), block the membrane with 5% bovine serum albumin and then incubated with the rabbit anti-Sp1 (PLA0044, SIGMA) and rabbit anti-cytochrome-P450 polyclonal antibody (PA1-343, SIGMA) followed by a peroxidase-conjugated anti-rabbit IgG antibody (A0545, SIGMA).

### 2.10. NCCR Sequencing

A nested-PCR was used to obtain the *NCCR* product for sequencing. Briefly, 100 ng of total DNA was amplified using two pairs of primers: first pair of primers, BKTT1 forward 5′ AAG GTC CAT GAG CTC CAT GGA TTC TTC C 3′ and BKTT2 reverse 5′ CTA GGT CCC CCA AAA GTG CTA GAG CAG C 3′, generating a 684 bp DNA fragment; the second pair of primers, BK-1 forward 5′ GGCCTCAGAAAAAGCTTCCACACCCTTACTACTTGA 3′ and BK-2 reverse 5′ CTTGTCGTGACAGCTGGCGCAGAA 3′, that amplified a portion of the first amplicon generating a fragment of 354 bp. The PCR products were purified using the PCR purification Kit (Qiagen, Hilden, Germany) and sequenced using the BigDye Terminator Cycle-Sequencing Ready Reaction (Applied Biosystems, Foster City, CA, USA). The sequences were analysed and edited using Bioedit 5.0.9 (Tom Hall of Ibis Therapeutics, Carlsbad, CA, USA).

### 2.11. Statistical Tests

The data were analysed using Student’s t-tests. *p*-values less than 0.05 were considered statistically significant.

## 3. Results

### 3.1. Rearranged NCCR-BKPyV Variants Show Higher Viral Loads But 10-Fold Lower miRNA Expression than Archetype NCCR BKPyV

**Figure 1:**
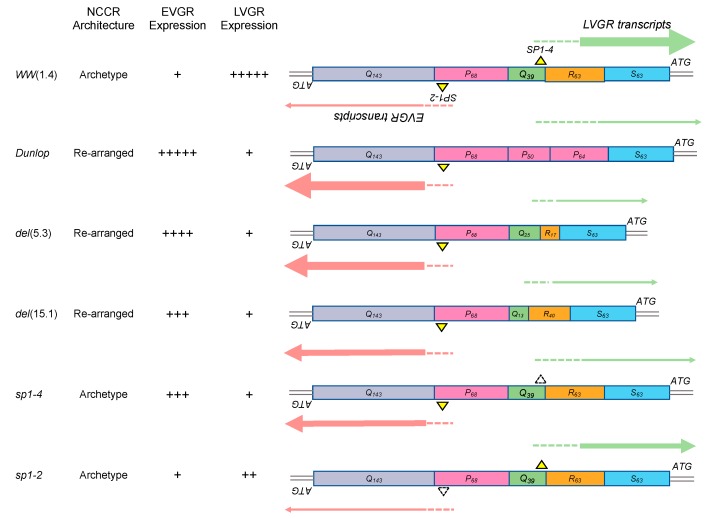
Schematic representation of *NCCR* architecture and *EVGR* and *LVGR* expression pattern of *BKPyV* strains. *BKPyV NCCR* architecture, Sp1 binding sites and expression levels are schematically summarized for the archetype (*WW1.4*), the laboratory-derived *rr-NCCR* (Dunlop), isolates from kidney transplant patients carrying rearranged *NCCRs: BKPyV rr-NCCR del*(*5.3*) and *del*(*15.10*) genome, as well as the BKPyV archetype NCCR carrying point mutations inactivating Sp1 binding to *LVGR*-proximal (*sp1-4*) and *EVGR*-proximal binding sites (*sp1-2*). Sp1 binding to the directional (non-palindromic) sequences is indicated as yellow triangle, dashed-line triangles indicate point mutations. The sequence blocks O, P, Q, R and S are indicated by coloured boxes and their respective length in base pairs in subscripts. The relative expression levels of the EVGR and the LVGR is represented by the thickness of the red and green arrows, whereby +++++, very strong; to + weak (see references [24,29,30]).

**Figure 2:**
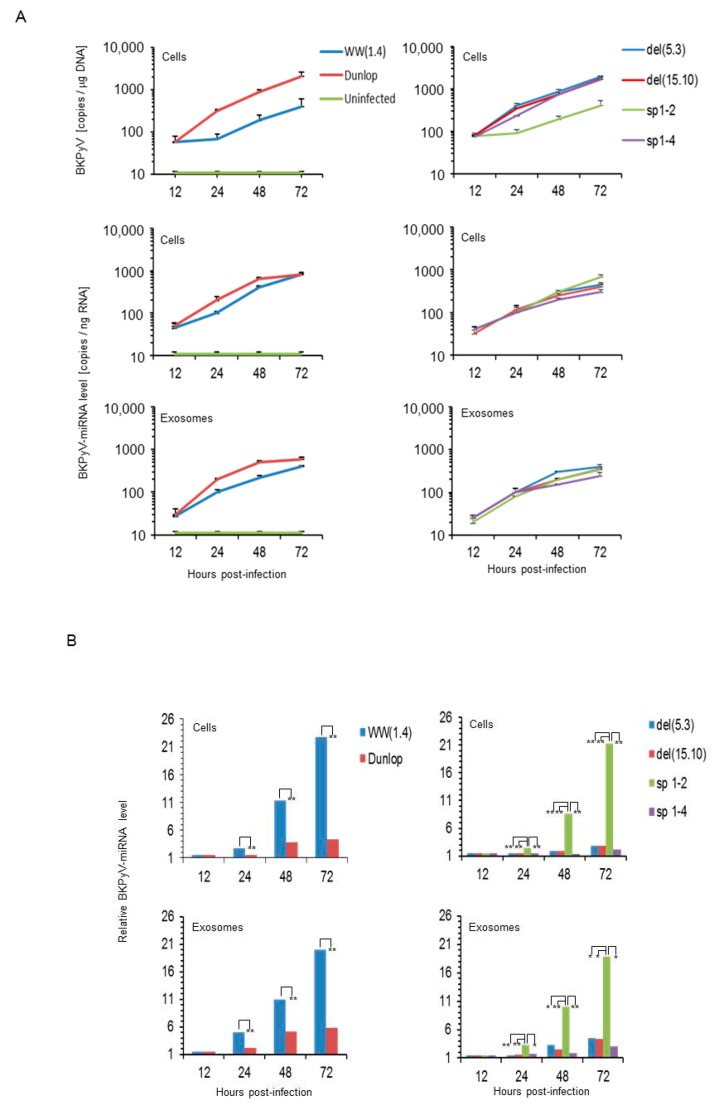
Viral replication and miRNA levels of archetype and different laboratory- or patient-derived *BKPyV* variants in cell culture. (**A**) Cos-7 cells were infected with the indicated strains (Figure 1) and viral load and BK-miRNA-5p expression were measured in cells and exosome preparations at 12, 24, 48 and 72 h post infection. *BKPyV* genome load was expressed per total µg DNA; miRNA given per total ng RNA (mean ± standard deviation of 3 independent experiments). (**B**) Time course comparing of miRNA expression levels in cells or exosome preparations following infection with archetype or indicated NCCR variant strains as described in Figure 1. Results are expressed as *BKPyV*-miRNA relative levels to DNA genome copy number normalized to archetype *WW*(*1.4*) clone values obtained at 12 h post-infection set to 1 (-fold; *, *p* < 0.05; ** *p* < 0.01).

To address the role of *NCCR* and microRNAs, we compared archetype and rearranged *NCCR-BKPyV* in cell culture. Accordingly, cloned genomes of the archetype *NCCR-BKPyV*-*WW*(*1.4*) and the laboratory stain *BKPyV*-Dunlop, which carries a *rr-NCCR* (Figure 1) were transfected into Cos-7 cells and infectious supernatants were prepared. Following infection, *BKPyV*-loads rapidly increased in Dunlop-infected cells but only slowly in archetype *WW*(*1.4*) infected cultures over 72h post infection (Figure 2A; left top panel). As shown previously [24,50,53], only one viral replication cycle is complete for the Dunlop strain under these conditions and only few secondary infection events have started as evidenced by large T-antigen positive/Vp1-negative immunofluorescence. *BKPyV* miRNA also increased during this time course but the levels seen in the archetype-infected cells (Figure 2A; left middle panel) and in the corresponding exosome preparations (Figure 2A; left bottom panel) were almost as high as in the Dunlop-infected cultures and therefore much higher than expected from the replication kinetics and the available genome copy number in particular.

Direct comparison of the miRNA levels expressed per *BKPyV* genome copy number revealed significantly higher expression in cells and in exosome preparations generated during archetype *BKPyV* infection than observed for the *rr-NCCR*-Dunlop strain (Figure 2B; left panels).

Given the difference in NCCR architecture between archetype *WW*(*1.4*) and rearranged laboratory Dunlop virus, we were interested to examine the patient-derived *rr-NCCRs del*(*5.3*) and *del*(*15.10*) variants that had emerged in kidney transplant recipients and had been shown to increase EVGR expression and replication rates in several previous studies [24,29]. The results demonstrated that both of patient-derived *rr-NCCR* variants behaved similar to the Dunlop-strain showing high replication levels and low miRNA expression in cells and exosome preparations (Figure 2A,B; right panels).

To investigate whether *NCCR* rearrangements or increased EVGR activity played a role in modulating the miRNA levels, we examined the (*sp1-4*)*NCCR-BKPyV*, which has an archetype architecture but shows increased EVGR expression and viral replication due to point mutations inactivating the *SP1-4* binding site upstream of the LVGR transcription start site [29,30] (Figure 1). As a control, we chose the (*sp1-2*)NCCR-BKPyV, in which the *SP1-2* site located upstream of the EVGR transcription start site was inactivated by point mutations and which showed only low EVGR expression and replication [29,30] (Figure 1). We observed high replication rates and low miRNA expression in cells and exosome-preparations for the (*sp1-4*)*NCCR-BKPyV*, whereas the opposite was true for (*sp1-2*)*NCCR-BKPyV* (Figure 2A,B; right panels). The results indicated lower miRNA expression was associated with increased EVGR expression and replication rates and that rearrangements of the *NCCR* were not necessary for the observed reduction in miRNA levels.

### 3.2. Sp1 Expression Levels Modulate BKPyV miRNA Levels in Archetype NCCR Virus And Variants With Low EVGR Expression But Not in Viral Variants Having an Activated EVGR Expression

Since the bi-directional expression of the archetype *NCCR* is critically modulated by Sp1 binding sites with high (*SP1-4*) and low affinity (*SP1-2*) binding sites in the *EVGR* and *LVGR* promoter, respectively [29,30], we wondered if knock-down of Sp1 had any influence on miRNA expression levels. Accordingly, Cos-7 cells were transfected with siRNA targeting SP1, with scrambled siRNA, or only mock-treated and miRNA expression levels were analysed after infection with archetype, the two point-mutant derivatives *sp1-2* and *sp1-4;* and the Dunlop strain (Figure 1).

**Figure 3:**
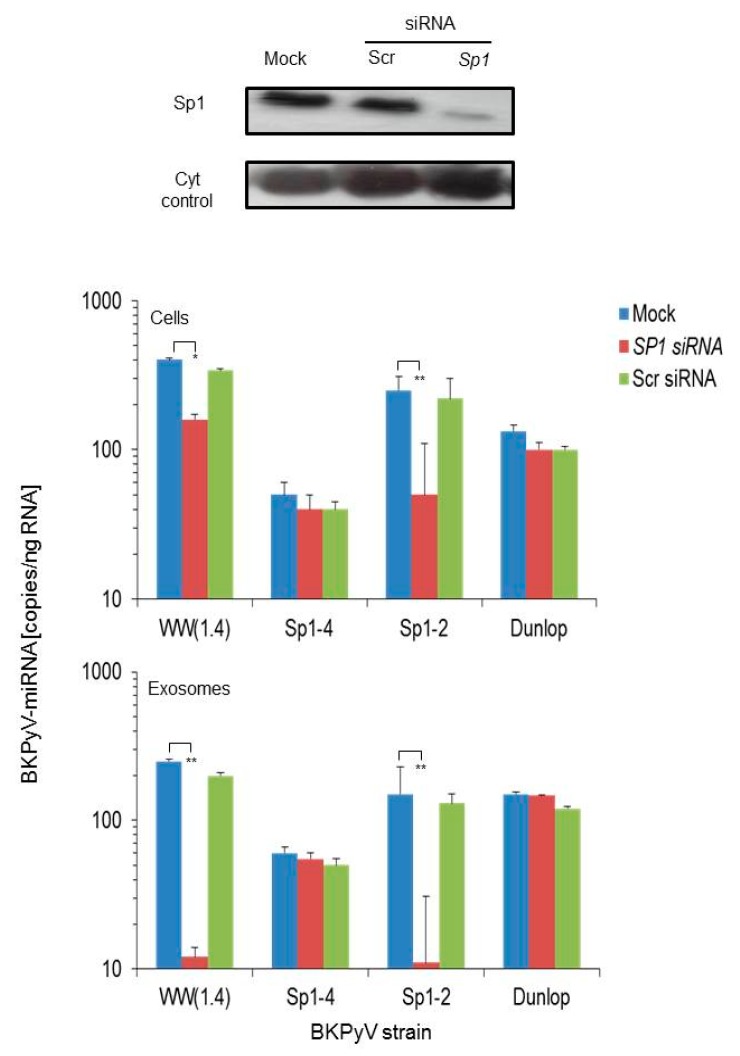
Effect of Sp1 knock-down on *BKPyV* miRNA-5p expression. Cos-7 cells were transfected with SP1-siRNA, scrambled Scr-siRNA or mock-treated and at 24 h posttransfection, infected with the indicated viral strains. Top panel shows the immunoblot of Sp1 and cytochrome P450 protein levels at 48 h post-transfection as described in Materials & Methods. Middle and lower panel shows the BK-miRNA-5p expression measured in cells and in exosome preparations at 48 h post infection, respectively. The values result from 3 independent experiments (mean ± standard deviation; Student’s t test, ** *p* < 0.01).

Sp1 knock-down was verified by immunoblotting demonstrating a significant reduction in the siRNA-*SP1*- but not in the scrambled siRNA- or the mock-treated control cells (Figure 3; top panel). In the mock and scrambled siRNA controls, the original observation was confirmed showing high levels of *BKPyV*-miRNA following archetype- and (*sp1-2*)*NCCR-BKPyV* infection, whereas low levels were seen following (*sp1-4*)*NCCR-BKPyV* and Dunlop infection, both in cells and corresponding exosome preparations (Figure 3; blue and green bars). In the siRNA-*SP1* treated cells, no change was seen in *BKPyV*-miRNA levels in Dunlop- or (*sp1-4*)*NCCR-BKPyV*-infected cells, whereas a significant reduction was seen in archetype and (*sp1-2*)*NCCR-BKPyV*-infected cells (Figure 3; red bars). The results indicated that knock-down of Sp1 protein by siRNA was associated with a significant reduction in cellular and exosomal miRNA levels in *BKPyV* strains having an intact *SP1-4* binding site, whereas there was little change in cells infected with virus strains showing constitutively activated EVGR expression. Thus, Sp1 availability for binding on the *SP1-4* site appeared to play a key role in *BKPyV*-miRNA expression levels.

### 3.3. BKPyV miRNA Levels in Primary Human Renal Tubular Epithelial Cells (RPTECs) Inversely Correlate with the Evgr-Activity and Replication Rate of Archetype and rr-NCCR-Viruses

**Figure 4:**
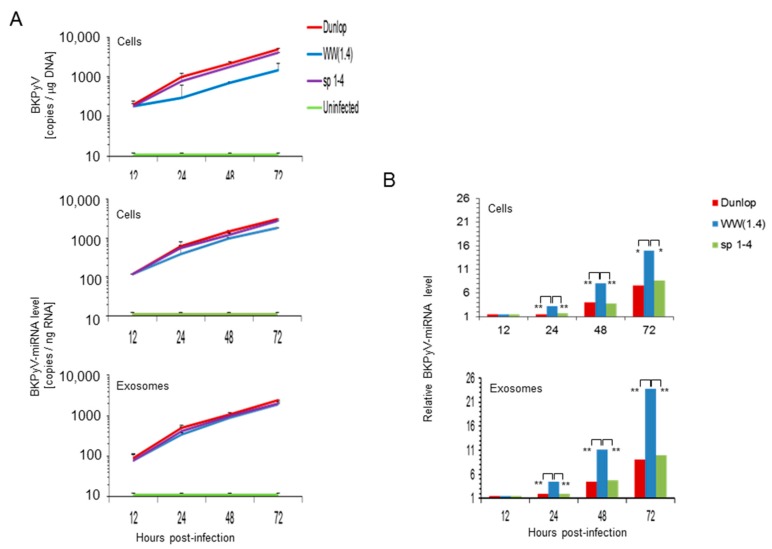
RPTECs infected with *BKPyV* variants having increased EVGR activity are associated with lower miRNA levels than archetype *BKPyV*. (**A**) Time course of *BKPyV* DNA replication and miRNA-5p expression in RPTECs and corresponding exosome- preparations. RPTECs were infected and viral DNA genome and *BKPyV*-miRNA-5p expression were measured at 12, 24, 48 and 72 h post infection as detailed in Materials & Methods. *BKPyV* genome load was expressed per total µg DNA; miRNA was quantified per total ng RNA (mean ± standard deviation of 3 independent experiments). (**B**) Comparison of relative miRNA expression levels per *BKPyV* genome copy (template) in cells. Results are expressed as *BKPyV*-miRNA levels normalized to *BKPyV* genome copy number, archetype *WW*(*1.4*) values at 12 h post-infection are set to 1 (-fold; * *p* < 0.05; ** *p* < 0.01).

To extend these studies to RPTECs, the natural target of *BKPyV* infection in the human host, these cells were infected with archetype *WW*(*1.4*)-, (*sp1-4*)- and Dunlop strains and *BKPyV*-DNA load and miRNA-5p expression was measured at 12, 24, 48 and 72 h post-infection. As described previously [29], *BKPyV*-Dunlop and the point mutant *sp1-4* replicated significantly faster than the archetype BKPyV-*WW(1.4*) but there was little difference in miRNA per total RNA in cells or exosome preparations (Figure 4A). However, the *BKPyV*-miRNA-5p levels per viral genome were significantly higher in the archetype-infected RPTECs and exosomes than in the corresponding preparations following Dunlop or (*sp1-4*)-infection (Figure 4B).

**Figure 5:**
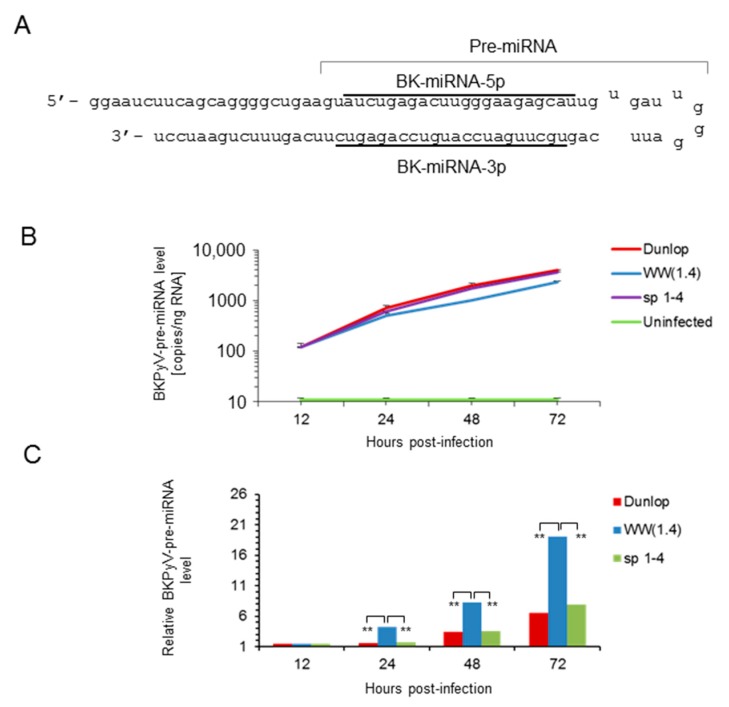
Time course of *BKPyV* pre-miRNA expression in infected RPTECs. (**A**) Schematic representation of the *BKPyV* pre-miRNA hairpin and the corresponding BKPyV-miRNA-5p and miRNA-3p sequence (underlined). (**B**) Time course of *BKPyV* pre-miRNA expression levels after infection of RPTECs with the indicated strains. Mean values ± standard deviation of 3 independent experiments are shown. (**C**) Comparison of relative *BKPyV* pre-miRNA expression levels per *BKPyV* genome copy (template) in cells. Results are expressed as *BKPyV*-miRNA levels normalized to *BKPyV* genome copy number, archetype *WW*(*1.4*) values at 12 h post-infection are set to 1 (-fold; ** *p* < 0.01).

To address the question, whether or not the differences in mature *BKPyV*-miRNA-5p were related to the expression levels of the miRNA gene or differences in maturation or degradation, we examined the amount of precursor miRNA (pre-miRNA) which consists of the 5′- and 3′-miRNA linked by a hairpin loop in RPTECs (Figure 5). The results indicated that the *BKPyV* pre-miRNA levels behaved similar to what has been observed for the mature *BKPyV*-miRNA-5p, namely being high in archetype virus but low in the variants (Dunlop and *sp1-4* mutant) having increased *EVGR* activity.

### 3.4. Pre-Infection Addition of BKPyV-Exosome Preparations Containing BKPyV miRNA Inhibit BKPyV Replication of Dunlop or sp1-4 Mutant But Have No Effect On Archetype BKPyV or sp1-2 Mutant Strains

Given the rapid replication of *BKPyV*-variants with activated EVGR expression having only low levels of BKPyV-miRNA per genome template, we wondered if rr-NCCR-BKPyV replication could still be down-regulated by miRNA. To this end, we transferred exosome preparations carrying BKPyV-microRNA cargo onto uninfected cells prior to BKPyV infection. To avoid potential interference or confounding factors associated with exosome preparations from BKPyV-infected cells, we prepared exosomes from Cos-7 cells transfected with the plasmid vector pcDNA6-BK-miRNA expressing BKPyV miRNA-5p and the pcDNA6-miRNA vector expressing an unrelated miRNA-5p as control (see Materials & Methods).

**Figure 6:**
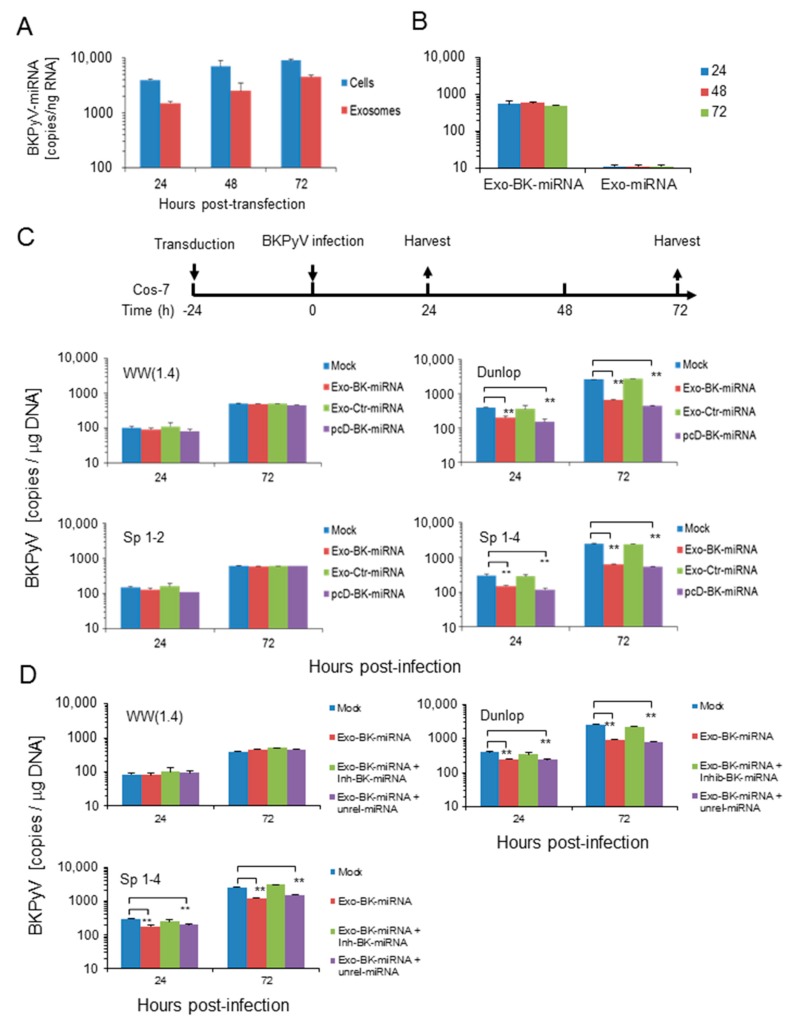
Addition of *BKPyV*-exosome preparations containing *BKPyV*-miRNA-5p before infection inhibits replication of *BKPyV* variants with activated *EVGR* expression (Dunlop and *sp1-4* mutant) but not archetype or (*sp1-2*) *BKPyV* replication. (**A**) Cos-7 cells were transfected with pcDNA6-BK-miRNA (pcD-BK-miRNA). Then, BK-miRNA expression in Cos-7 cells and in exosomes enriched vesicles derived from supernatant was measured after indicated time post transfection. (**B**) Detection of *BKPyV*-miRNA-5p in Cos-7 cells after addition of exosome preparations from pcDNA6-BK-miRNA-5p and control transfected or mock-treated cells. (**C**) *BKPyV* loads after exosome addition prepared from cells transfected with the indicated expression vectors (Exo-BK-miRNA, unrelated miRNA, or mock), or after direct transfection of the pcDNA6-BK-miRNA expression vector (see flow-chart). (**D**) *BKPyV* loads after exosome addition prepared from cells transfected with the indicated expression vectors (Exo-BK-miRNA, unrelated miRNA, in the presence or absence of a specific antago BK-miRNA called Inh-BK-miRNA), or after direct transfection of the pcDNA6-BK-miRNA expression vector (see flow-chart). The corresponding exosome preparations are indicated according to the transfection vector (e.g., BKPyV-miRNA-5p; unrelated miRNA; mock; in the presence of Inh-BK-miRNA, a synthetic phosphoro-thioate oligonucleotide with the sequence complementary to the BK-miRNA or a corresponding unrelated miRNA. The values represent the mean ± standard, ** *p* < 0.01.

The time course up to 72 h post-transfection showed increasing BKPyV-miRNA-5p levels in cells and in corresponding exosome preparations (Figure 6A). Exosome uptake and internalization after addition on Cos-7 cells was verified by the positive fusion assay performed based on octadecyl-rhodamine lipid probe dequenching as previously described (Supplement Figure S2). The amount of detectable BKPyV-miRNA-5p after exosome addition did not change up to 72 h post-addition, whereas no BKPyV-miRNA was detected when using exosomes from control-transfected cells (Figure 6B).

We next tested the effect of exosome preparation on subsequent viral infection. The results indicate that replication of the *BKPyV*-Dunlop and the point mutant (*sp1-4*)-*NCCR-BKPyV* was significantly inhibited, if infection was performed after addition of exosomes prepared from *BKPyV*-miRNA-5p transfected cells. Instead, exosomes prepared from cells transfected with an unrelated miRNA expression vector or from untransfected cells (mock) had no effect (Figure 6C; top panels). Under these conditions, archetype *BKPyV* and the (*sp1*-2)-point mutant replicated slowly but without significant inhibition by exogenous exosome addition. Of note, no inhibition of *BKPyV*-Dunlop or *sp1-4* mutant replication was observed, when exosome preparations carrying *BKPyV*-miRNA-5p and a specific antago-BK-miRNA (Inh-BK-miRNA) molecules, whereas unrelated synthetic miRNA (Unrel-miRNA) had no effect (Figure 6D; bottom panels).

### 3.5. Analysis of the BKPyV NCCR Architecture and Exosomes Content in Urine Samples of Immunocompromised Patients

**Figure 7:**
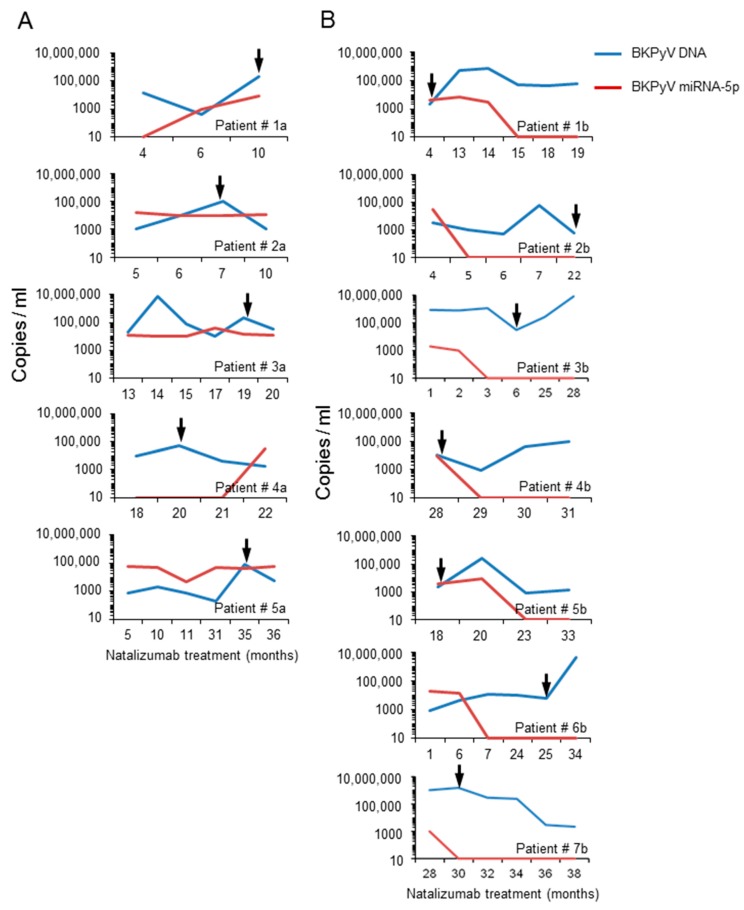
*BKPyV* DNA viral load and miRNA-5p expression in urine samples from multiple sclerosis patients. Urine sample were analysed over indicated time of follow-up and at the indicated time points, urine *BKPyV* loads and miRNA levels in exosome preparations were determined. Arrows indicate the time at which *NCCR* sequence was obtained from urine samples. Panels on the left show the results obtained for 5 patients with *BKPyV* carrying archetype *NCCRs* (patient ID 1a–5a); Panels on right show those with rearranged *NCCR-BKPyV* (patient ID 1b–7b).

To explore the potential relevance in humans, urine samples from 12 natalizumab-treated multiple sclerosis patients were analysed. Twelve out of 25 *BKPyV*-DNA positive patients were identified among 42 natalizumab-treated multiple sclerosis patients enrolled in previous study [47]. The sequencing of the *BKPyV NCCRs* identified the presence of predominantly *rr-NCCR* in the urine samples collected from 7 patients, subjected to long term natalizumab treatment. Archetype NCCR *BKPyV* strains were detected in the urine samples from the remaining 5 patients. Overall, the urine *BKPyV* load in patients with predominantly *rr-NCCR BKPyV* was higher than in patients with archetype *BKPyV*. The opposite was true for *BKPyV* miRNA-5p expression levels, being low in the 7 patients carrying *rr-NCCR* variants and in the 5 patients shedding archetype *NCCR BKPyV* (Figure 7).

## 4. Discussion

The regulation of *BKPyV* persistence and reactivation is receiving increasing attention [8] in the light of the almost universal but well controlled *BKPyV* infection of the general human population and the increasing *BKPyV* diseases in patients under potent immunosuppressive regimens [4,5,54,55]. So far, two major mechanisms of regulating *BKPyV* replication have emerged, which involve transcriptional, *NCCR*-based and post-transcriptional miRNA-based mechanisms targeting EVGR expression [24,41]. Pioneering work from Imperiale and co-workers has demonstrated that both layers of regulation appear to interact since low *BKPyV* miRNA-5p levels were seen in infection by rapidly replicating *BKPyV* carrying *rr-NCCRs*, whereas high miRNA-5p levels were seen in archetype *BKPyV* [41].

In this study, we extend these observations from laboratory strains to patient-derived isolates and show that *BKPyV* miRNA-5p levels are decreased following infection of patient *BKPyV* carrying diverse *NCCR* rearrangements (Figure 1). Moreover, we demonstrate that point mutations inactivating a single Sp1 binding site (*sp1-4*) in an otherwise archetype *NCCR* have a similar effect. This (*sp1-4*)-mutation has been shown to confer a phenotype of increased EVGR expression and more rapid viral replication (group 1) similar to *rr-NCCR BKPyV* variants [29]. Thus, our results indicate that NCCR rearrangements are not required for the lowered miRNA levels but suggest that the activation of EVGR expression is important. The latter was supported by another Sp1 point mutant (*sp1-2*) showing which permits only modest EVGR expression, while LVGR expression is also reduced (group 3) [29] and which showed high-levels of BKPyV miRNA similar to the archetype *WW*(*1.4*)-*NCCR-BKPyV*. Of note, the *BKPyV* miRNA-5p expression of archetype and (*sp1-2*)-mutated *NCCR-BKPyV* could be significantly diminished by reduced Sp1 levels following siRNA-*SP1* knock-down. Conversely, variants carrying rearranged or (*sp1-4*)-mutated *NCCRs* were not affected. Parallel experiments in human RPTECs generated similar results, suggesting that the observations were also relevant in the human situation. The data support the notion that *BKPyV* miRNA-5p levels and the *NCCR* activity are critically linked by Sp1 binding to the *SP1*-4 site. Independently, a principle role of Sp1 for *BKPyV* replication has recently emerged from a genome-wide interference study [56].

While our study provides a detailed accounting of the inverse association of EVGR activity and miRNA-5p expression, the exact molecular mechanisms need to be addressed further. Inverting the orientation of the *rr-NCCR* has been shown to confer high LVGR expression at the expense of EVGR [23,24] and corresponding recombinant viruses show increased miRNA expression [41]. Whether such LVGR transcription opens the access to the miRNA promoter region, provides transcription factors and enzymatic complexes, or extended transcripts is presently discussed. Conversely, activated EVGR transcription might simply outcompete the available miRNA transcripts, or confer resistance to downregulation by another as yet unknown mechanism including antisense stealth transcripts or processing from pre-miRNA to miRNA. In our study, we observed that *BKPyV* pre-miRNA were similarly affected by the *NCCR* activity as the mature miRNA-5p suggesting that transcript generation rather than miRNA processing, maturation or degradation was affected. No evidence for a difference in cellular miRNA by packaging and secretion as exosomes was obtained, since both cellular and exosomal levels mirror-imaged the activity of the NCCR in both compartments. The possibility that *BKPyV* miRNAs were no longer able to downregulate replication of variants with activated EVGR was refuted in time course experiments adding *BKPyV*-miRNA-5p loaded exosome preparations to host cells prior to infection, which demonstrated a significant reduction of the *BKPyV*-Dunlop and the (*sp1-4*)-point mutant. This interpretation was supported by inclusion of an antagonist synthetic phosphorothioate oligonucleotide reversing replication inhibition.

The polyomavirus miRNA-5p has been proposed to act as an important safeguard silencing residual large-T-antigen expression of the archetype *BKPyV* during viral persistence [34,41]. Efficient transcriptional and posttranscriptional synergy in downregulation of large T-antigen expression may permit escape from cytotoxic CD8 T-cell effectors, which we have been shown to preferentially target T-antigen epitopes [38,39]. Conversely, a bi-directional link between *NCCR* activity and miRNA-expression appears biologically plausible, when signals of activating EVGR expression are sensed in the latently infected host cell, for example by displacing Sp1 from the *SP1-4* binding site in the LVGR promoter [30]. Downregulating the posttranscriptional miRNA then permits for an efficient progression through the viral life cycle. In immunosuppressed patients lacking sufficient CD8 T-cell activity, the high and prolonged viral replication allows for the emergence of *rr-NCCR* variants conferring an activated EVGR expression and high-replication capacity [24], while reducing posttranscriptional interference through BK-miRNA-5p downregulation

Given the potential clinical relevance for immunocompromised patients, we explored *BKPyV* shedding, *NCCR* architecture and miRNA levels in multiple sclerosis patients treated with natalizumab. We found that patients shedding *BKPyV* with *rr-NCCRs* had on average higher urine viral loads but lower miRNA-5p levels in the exosome-enriched vesicles. Conversely, patients shedding *BKPyV* with the archetype *NCCR* architecture showed lower viral loads and typically higher miRNA-5p levels in urinary exosome preparations. Thus, these preliminary data, if confirmed, seem to be consistent in their almost dichotomous nature and also provide an incentive for further work, which may be of relevance for clinical diagnostic and therapeutic approaches [42,44].

**Figure 8:**
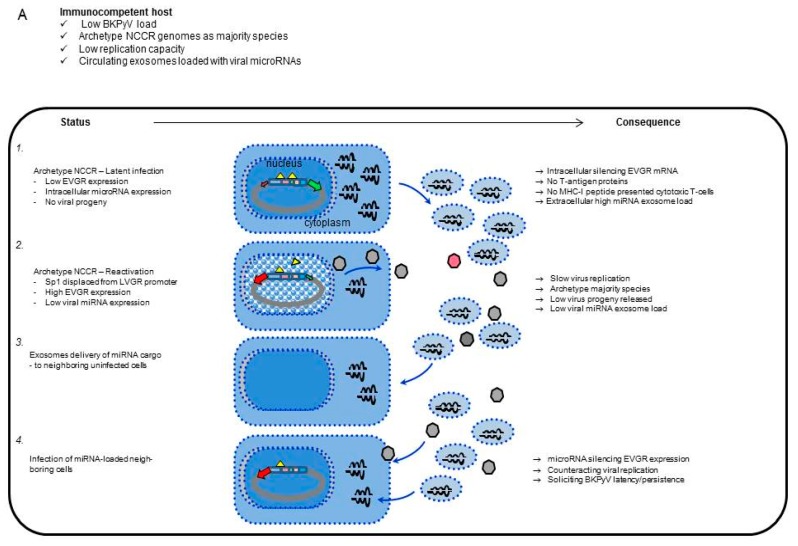

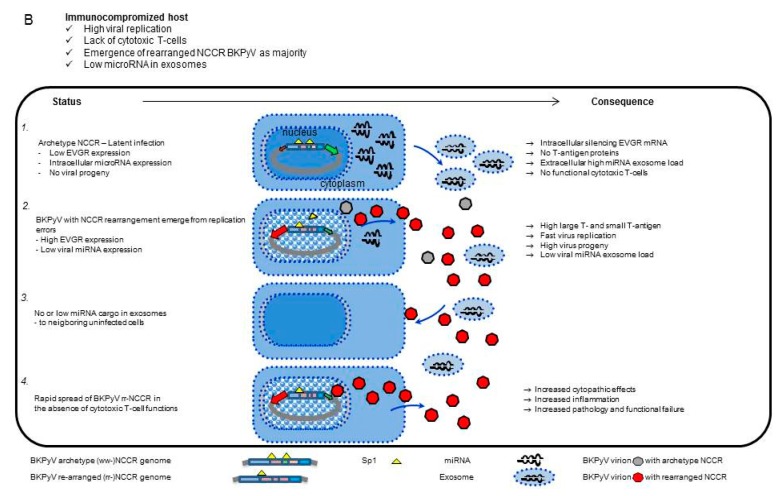
Model of BKPyV miRNAs expression and exosome traffic. (**A**) Immunocompetent host:1- Archetype *NCCR-BKPyV* establishes latent infection with low EVGR expression and strong silencing by high-level miRNA expression; 2- Displacement of Sp1 from *SP1-4* through host cell activation permits activation of EVGR expression and lowering of miRNA expression; 3- Exosomes deliver *BKPyV* miRNA cargo to uninfected cells; 4- Subsequent infection with archetype BKPyV counteract new rounds of viral replication, thereby avoid recognition by cytotoxic T-cells. (**B**) Immunocompromised host: 1- Status and consequences during *BKPyV* archetype persistent infection; 2- *BKPyV* with rearranged *NCCR* emerge in immunocompromised patients as the immune selection pressure fades; 3- No or low miRNA cargo in exosome delivery to uninfected neighbouring cells; 4- Rapid spread, increased cytopathology and functional failure.

Finally, there is increasing evidence reported that other viruses including those that have the propensity to establish latent/persistent infections such as herpesviruses use miRNA regulation not only intracellularly but also in exosomes [57]. Thus, besides a principle role in virus biology, our data, together with those of other researchers, strongly suggest that viral miRNA should be explored further in a virological and clinical context. To integrate our results into the work of other researchers and to stimulate the corresponding projects, we present a model (see Figure 8) in which host cell signals, viral *NCCR* activity and miRNA expression permit fine tuning of persistence, reactivation of replication, spread to neighbouring host cells, for example, in the epithelial monolayer of the renal tubules and hiding from cytotoxic large T-antigen-specific CD8 T-cells, unless the host is immunocompromised. The potential role of exosomes and miRNA cargo in cell to cell communication offers an interesting possibility that could potentially be harnessed for antiviral therapy.

## Supplementary Materials

The following are available online at http://www.mdpi.com/1999-4915/10/9/466/s1, Figure S1: Size, concentration and markers of Cos-7 exosome enriched vesicles., Figure S2: Cells adsorption and penetration of exosomes enriched vesicles containing *BKPyV* miRNAs.
