# Supplementary figures and images for: BK Polyomavirus MicroRNA Levels in Exosomes Are Modulated by Non-Coding Control Region Activity and Down-Regulate Viral Replication When Delivered to Non-Infected Cells Prior to Infection
Source: Viruses. 2018 Aug 30;10(9):466. doi: 10.3390/v10090466 (PMC6164188; doi:10.3390/v10090466)

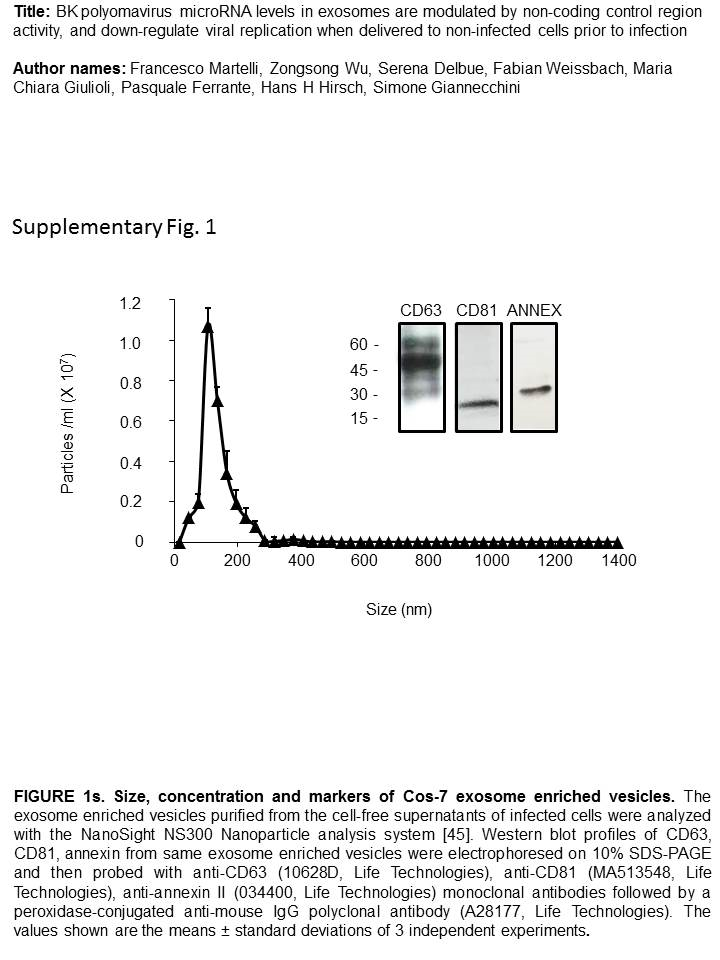

Supplement: Supplementary file 1 [file viruses-10-00466-s001.zip › Figures 1-2s supplementary/Figure 1s Supplementary.tif]

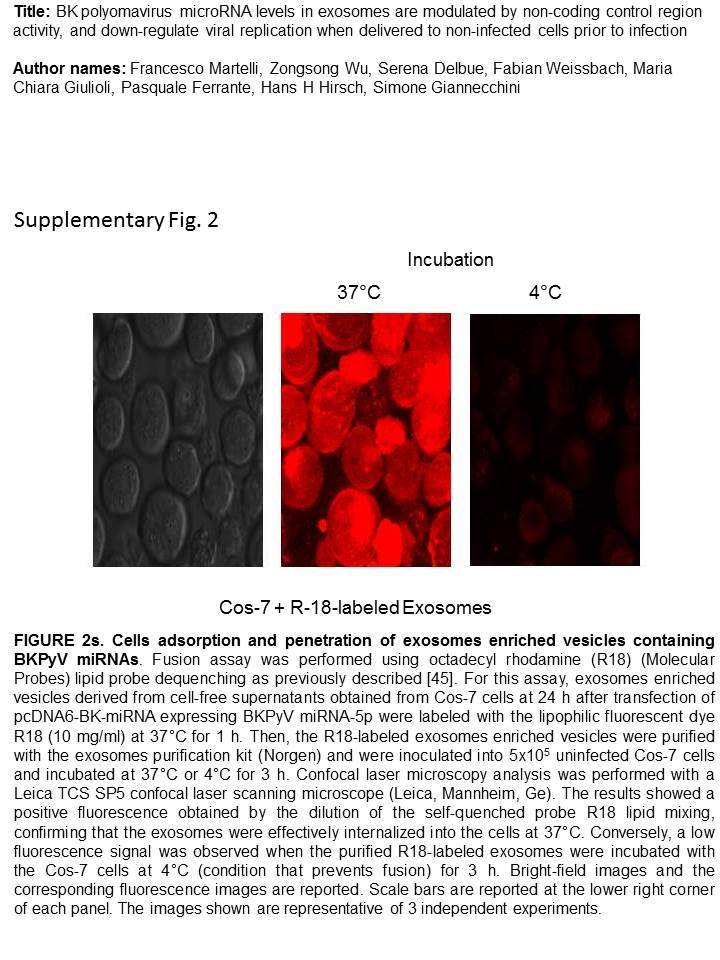

Supplement: Supplementary file 1 [file viruses-10-00466-s001.zip › Figures 1-2s supplementary/Figure 2s Supplementary.tif]
